# Comparison of Tracheal Wash and Bronchoalveolar Lavage Cytology in 154 Horses With and Without Respiratory Signs in a Referral Hospital Over 2009−2015

**DOI:** 10.3389/fvets.2018.00061

**Published:** 2018-03-26

**Authors:** Heini Rossi, Anna-Maija Virtala, Marja Raekallio, Emmi Rahkonen, Minna M. Rajamäki, Anna Mykkänen

**Affiliations:** ^1^Department of Equine and Small Animal Medicine, Faculty of Veterinary Medicine, University of Helsinki, Helsinki, Finland; ^2^Veterinary Biosciences, Faculty of Veterinary Medicine, University of Helsinki, Helsinki, Finland

**Keywords:** equine, bronchoalveolar lavage, tracheal wash, lung, respiratory

## Abstract

Most equine lower respiratory diseases present as increased airway neutrophilia, which can be detected in tracheal wash (TW) or bronchoalveolar lavage fluid (BALF) cytology samples. The aim was to compare the TW and BALF results in a population of client-owned horses with and without clinical respiratory disease signs. A secondary aim was to determine the sensitivity (Se) and specificity (Sp) of TW and BALF neutrophilia in detecting respiratory disease. The cutoff values for neutrophils were also evaluated. Retrospective data from 154 horses of various breeds that had been subject to both TW and bronchoalveolar lavage (BAL) sampling at rest during 2009−2015 were used. The horses were divided into three groups based on the presenting signs, physical examination, and endoscopy mucus score. Neutrophil counts of >20% in TW and >5% in BAL were considered abnormal. Cytology results between groups, correlations between TW and BALF cell types, and tracheal mucus score were analyzed. Two graph receiving operating characteristic (ROC) curves of the neutrophil percentage values of TW and BALF were created to determine the optimal cutoff values and to calculate the diagnostic Se and Sp for diagnosing airway inflammation in horses with and without clinical respiratory signs. The Se and Sp of TW and BALF neutrophil percentages were further estimated using a two-test one-population Bayesian latent class model. The two tests showed substantial agreement, and only 17.5% of the horses were classified differently (healthy vs. diseased). The neutrophil percentage was found to correlate between TW and BALF. The Se and Sp of TW were generally higher than for BAL when estimated with area under the curve or Bayesian model. Cutoff values of 17.7% for TW and 7% for BALF were indicated by the ROCs. We conclude that TW is a more sensitive and specific method in our patient population. We suggest that the current neutrophil cutoff values of 20% for TW and 5% for BALF would still be appropriate to use in clinical diagnosis of airway inflammation. However, further studies with other cell types and in other populations are warranted to determine the best sampling method for individual horses.

## Introduction

Cytological analysis of tracheal wash (TW) and bronchoalveolar lavage fluid (BALF) is a common diagnostic procedure in equine practice when assessing the lower respiratory tract health of the horse (1, 2). The choice of the sampling method is related to the suspected disease process. TW is considered to give a better representation of the whole lung than bronchoalveolar lavage (BAL) and, therefore, often preferred in cases where an infectious disease is suspected because secretions from the affected lung areas will collect in the trachea (3). The advantages of TW in clinical practice are its lower costs and the potential avoidance of doping withdrawal times that arise from sedation and local anesthesia that are often administered during the BAL procedure to restrain the horse and suppress coughing (3). On the other hand, BAL is recommended in suspected diffuse non-bacterial lung disease, and it is considered to be a more sensitive technique for detecting lower airway inflammation (2, 4). Furthermore, BALF cytology may correlate better with lung histopathology results (4). In cases of uncertain etiology, performing both diagnostic tests simultaneously has been suggested in order to achieve accurate diagnosis (3, 5).

Chronic lower airway inflammation in horses presents as two phenotypes: milder inflammatory airway disease (IAD) and more severe recurrent airway obstruction (RAO). Both IAD and RAO are considered to comprise the equine asthma syndrome (4, 6, 7). Equine asthma presents as neutrophilic inflammation of the lungs, although in mild asthma, eosinophilic and metachromatic disease phenotypes have also been recognized (4). Asthmatic horses have a chronic cough, nasal discharge, and exercise intolerance (4). The horses with severe asthma present with increased respiratory effort (4).

In addition to chronic respiratory signs, clinical diagnosis of equine asthma is often based on increased amount of tracheobronchial mucus and inflammatory cells in the respiratory tract samples (2, 4). Most importantly, the elevated proportion of neutrophils in either TW or BAL fluid (BALF) is considered to indicate airway inflammation, and cutoff values for neutrophil percentage have been set at 20% for TW and 5% for BAL (3, 4). A 1% cutoff value for eosinophils in TW or BALF cytology and the cutoff of 2% for metachromatic cells in BALF cytology have also been considered (3, 4). Recently, new cutoff values of either >10% neutrophils, >5% mast cells, or >5% eosinophils for BALF cytology have been proposed for the diagnosis of airway inflammation in order to allow more variability caused by the variation in sampling techniques, which still need to be standardized in horses (4, 8–11). Moreover, a consensus for a true gold standard for diagnosing equine airway inflammation is nevertheless still missing, and the suggested cutoff values are largely based on observational studies and expert opinion. To our knowledge, the sensitivity (Se) and specificity (Sp) of TW and BALF cytology have not been assessed in the diagnosis of respiratory disease.

In most previously published studies, TW and BALF cytology results have shown poor correlation (5, 12–14). In contrast, Winder et al. (15, 16) reported correlations between the neutrophil percentages in TW and BALF samples. However, to our knowledge, there are no recent studies published that compared TW and BALF cytology among horses of varying ages and disciplines that have naturally occurring respiratory disease. Therefore, the first aim of this study was to compare the two airway sampling methods in a population of client-owned horses with signs related to respiratory disease and in horses with no clinical respiratory signs. Due to the lack of previous studies regarding the Se and Sp of TW and BALF cytology, a secondary aim was to evaluate the cutoff values for neutrophils in TW and BALF in order to justify their use in clinical practice.

## Materials and Methods

### Animals

All horses (*n* = 154) that had been subject to both TW and BAL sampling at rest during 2009−2015 in Helsinki University Equine Teaching Hospital were included in this retrospective study. The horses were client-owned and brought to the hospital for investigation of the presenting problem. When possible, the owners were requested to bring an asymptomatic companion horse for research purposes. Prior to respiratory endoscopy and sampling, all horses underwent physical examination and hematology analysis. The horses were divided into three groups based on the clinical signs and endoscopy findings. Group 1 included clinically healthy control horses without signs potentially related to respiratory disease (*n* = 33) that were sampled by TW and BAL methods for research purposes with the owners’ informed written consent. Thirteen of these horses were from the same properties than the symptomatic horses. Group 2 included horses that had at least one of the following signs: cough, nasal discharge, epistaxis, fever or poor performance, and did not belong to either Group 1 or Group 3 (*n* = 79). Group 3 included horses that had both cough and nasal discharge at the time of the examination and/or highest mucus score (3/3) during endoscopy that confirmed respiratory disease (*n* = 42). Only one horse had mucus score 3/3 without clinical signs reported by the owner. Retrospective patient data were used to identify and categorize the symptomatic horses, and the data were deidentified prior to use. The study was carried out in accordance with the recommendations of the Finnish Animal Experiment Board (asymptomatic horses) and Viikki Ethics Committee (symptomatic horses). The protocol was approved by the Finnish Animal Experiment Board.

### TW Collection

Horses were restrained in stocks and sedated using intravenous detomidine (0.005−0.01 mg/kg, Domosedan^®^, Oriola, Espoo, Finland) and butorphanol (0.005−0.02 mg/kg, Butodol^®^, MSD Animal Health, Boxmeer, Netherlands). A twitch was used to restrain the horse when needed. Horses were sampled without prior exercise (at least 12 h). A 220 cm long, 11 mm diameter video endoscope (Pentax, Tokyo, Japan) was passed *via* the ventral nasal meatus to the pharynx and then advanced into the trachea *via* the rima glottidis. The amount and nature of the tracheal mucus deposits were observed and graded semiquantitatively as 0 (none), 1 (mild), 2 (moderate), and 3 (marked). The endoscope was advanced to the mid-cervical trachea, and 10−20 mL of sterile 0.9% saline at room temperature was injected through either a single lumen polypropylene catheter or a double lumen catheter (Mila International, Erlanger, KY, USA) that was advanced to the trachea through the biopsy channel of the endoscope. After saline injection, the endoscope was advanced until the curvature of the distal trachea and the deposit of the mucus and saline were observed at the site of the thoracic inlet. As much fluid as possible was aspirated *via* the catheter. Samples were submitted for the laboratory analysis and were processed within 15 min of collection.

### Bronchoalveolar Lavage

Immediately following the transendoscopic TW procedure, BAL was performed. The endoscope was advanced further to the tracheal bifurcation, and 40−60 mL of 1% lidocaine or mepivacaine was injected onto the carina and the right mainstem bronchi to decrease coughing. The endoscope was advanced further to the right caudodorsal lobe until significant resistance was noted, and the endoscope was wedged into a small bronchus. Then, 240−360 mL of sterile 0.9% isotonic saline at room temperature was injected through the biopsy channel in one single volume followed by immediate aspiration. Syringes with the retrieved BALF samples were immediately placed on ice and submitted to laboratory analysis within 30 min of their collection. Samples were considered adequate when they contained a foamy surfactant layer. No samples had to be discarded due to inadequate quality.

### Cytological Analysis of TW and BALF Samples

Slides of the TW samples were prepared for differential cell counts by centrifugation of undiluted samples and subsequent smear of the cell pellet. Pooling the fluid from the syringes and cytocentrifugation (Thermo Scientific Cytospin 4 centrifuge; Thermo Fisher Scientific, Waltham, MA, USA) of undiluted samples was used for the BALF. All slides were stained with the May–Grünwald–Giemsa stain. A single experienced blinded person performed the differential counts of inflammatory cells by counting 300 cells from both TW and BALF slides. The analysis results for each cell type were expressed as a percentage of total cells.

### Statistical Analyses

Statistical analyses were performed using the statistical software SPSS 22.0 (IBM SPSS Statistical Package version 22, New York, NY, USA). A Kolmogorov–Smirnov test and Q–Q plots were used to assess the normality of the data. For most variables, the data were not normally distributed; therefore, non-parametric tests were used. Differences between the three groups for age and TW and BALF cytology were analyzed using a Kruskal–Wallis one-way analysis of variance, and for pairwise comparisons Dunn’s procedure (17) with a Bonferroni adjustment was used. Correlations between the tracheal mucus score and the different cell types for TW and BALF were examined by calculating the Spearman’s correlation coefficient with a Bonferroni adjustment for multiple comparisons. Differences were considered significant at *P* < 0.05.

### Evaluation of TW and BAL Test Results for Neutrophils

The testing was performed using the currently accepted cutoff values for neutrophil percentage where >20% in TW and >5% in BALF were considered abnormal (3, 4). The Fisher’s exact test was used for comparison of the TW and BAL test results (i.e., diseased or non-diseased as based on the cutoffs) for all horses. A kappa value was calculated using EpiTools^1^ and followed the guideline values for interpretation: ≤0 poor, 0.01–0.2 slight, 0.21–0.4 fair, 0.41–0.6 moderate, 0.61–0.8 substantial, and 0.81–1 almost perfect to perfect agreement (18). The kappa value was then used to compare the agreement between the two sampling procedures in the population that were beyond chance. Two graph receiving operating characteristic (ROC) curves were created and the area under the curve (AUC) calculated with EpiTools (see text footnote 1) for neutrophil percentage values in Group 1 (asymptomatic) and Group 3 (most severe signs) to find the optimal cutoff values (Youden’s *J* value) for TW and BALF within this population of horses and the corresponding diagnostic Se and Sp values to distinguish non-diseased from the diseased. For the interpretation of the AUC, the following guideline values were used (19): 0.5 non-informative, 0.5 < AUC ≤ 0.7 less accurate, 0.7 < AUC ≤ 0.9 moderately accurate, 0.9 < AUC < 1 highly accurate, and AUC = 1 perfect test. The Fisher’s exact test was rerun with the optimal cutoff values obtained from the ROCs for only Group 2 (the most heterogeneous group) for comparison of the TW and BAL test results (diseased or non-diseased based on the cutoff values).

Due to the lack of a true gold standard for evaluating equine airway inflammation, Se and Sp of TW and BALF neutrophil percentages were further estimated by a two-dependent test one-population Bayesian latent class model (20). The model allowed for a conditional dependence between TW and BAL results, since both TW and BAL samples were obtained from the airways of same animals, and they were considered related. The model was run in OpenBUGS© 3.2.3 rev 1012^2^ using code described in Branscum et al. (21) and available elsewhere.^3^ The model requires informative prior data for at least one test to ensure adequate identification. Expert opinions of the first and last authors were used for estimating the prior values for prevalences in the two populations (Group 1 and Group 3) and for Se and Sp of the TW test. The used mode estimates for prevalence (p) of true respiratory disease among the studied populations of horses were for the diseased population (Group 3) p1 95% (95% sure > 90%) and for the non-diseased population (Group 1) p2 10% (95% sure < 30%). The used estimates for Se and Sp to differentiate diseased from non-diseased for the TW test were Se1 mode 95% (95% sure > 90%) and Sp1 mode 70% (95% sure > 60%) for diseased population and Se2 mode 85% (95% sure > 70%) and Sp2 mode 90% (95% sure > 80%) for non-diseased population. The corresponding *a* and *b* values for beta-distributions were calculated using epi.betabuster^4^ in R© 3.3.0^5^ (R 2016) and were as follows: p1*a* = 99.70, p1*b* = 6.19; p2*a* = 2.08, p2*b* = 10.73; Se1*a* = 99.70, Se1*b* = 6.19; Sp1*a* = 47.53, Sp1*b* = 20.94; Se2*a* = 23.90, Se2*b* = 5.04; and Sp2*a* = 42.57, Sp2*b* = 5.62. Non-informative [beta (1,1)] priors, which allowed all values between 0 and 1 to have equal probability, were used for test 2 (BAL). After discarding the first 5,000 samples as burn-in, the next 150,000 iterations for inferences about Se, Sp, and prevalence were used. The model convergence was assessed by visual inspection of trace plots and running three chains from dispersed initial values.

## Results

The median age of all horses (*n* = 154) was 10 years (range 1–26 years). Breeds included the Finnhorse (*n* = 48), other Coldblood (*n* = 10), Warmblood (*n* = 41), Standardbred (*n* = 35), and pony (*n* = 20). Of these horses, 121 had one or several acute or chronic signs potentially related to respiratory disease including cough (*n* = 69), nasal discharge (*n* = 43), fever (*n* = 2), epistaxis related to exercise (*n* = 8), or poor performance (*n* = 66). Based on the history, clinical examination, airway endoscopy, and sample cytology, most horses of Group 3 were diagnosed with equine asthma [severe asthma (RAO; *n* = 25) and mild asthma (IAD; *n* = 7)], exercise induced pulmonary hemorrhage (EIPH; *n* = 1), other diagnosis related to respiratory tract (*n* = 8), and undetermined diagnosis (*n* = 1). The seventy-nine horses in Group 2 had varying diagnoses including mild asthma (*n* = 40), severe asthma (*n* = 13), EIPH (*n* = 2), other diagnosis related to respiratory tract (*n* = 8), other diagnosis not related to respiratory tract (*n* = 6), and undetermined diagnosis (*n* = 10).

**Figure 1:**
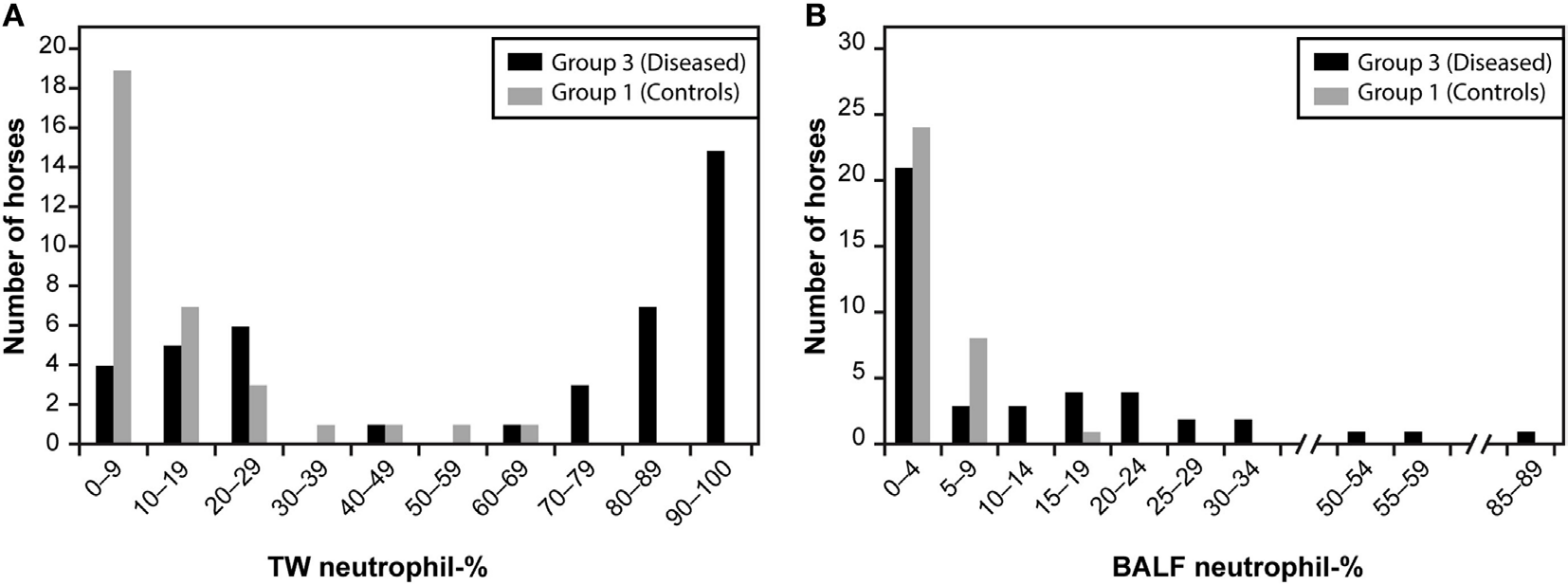
The tracheal wash [TW; **(A)**] and bronchoalveolar lavage fluid [BALF; **(B)**] neutrophil percentage histograms among Group 1 and Group 3 horses. Group 1 included clinically healthy control horses without signs potentially related to respiratory disease (*n* = 33), and Group 3 included horses that had both cough and nasal discharge and/or highest mucus score (3/3) confirming respiratory disease (*n* = 42).

**Figure 2:**
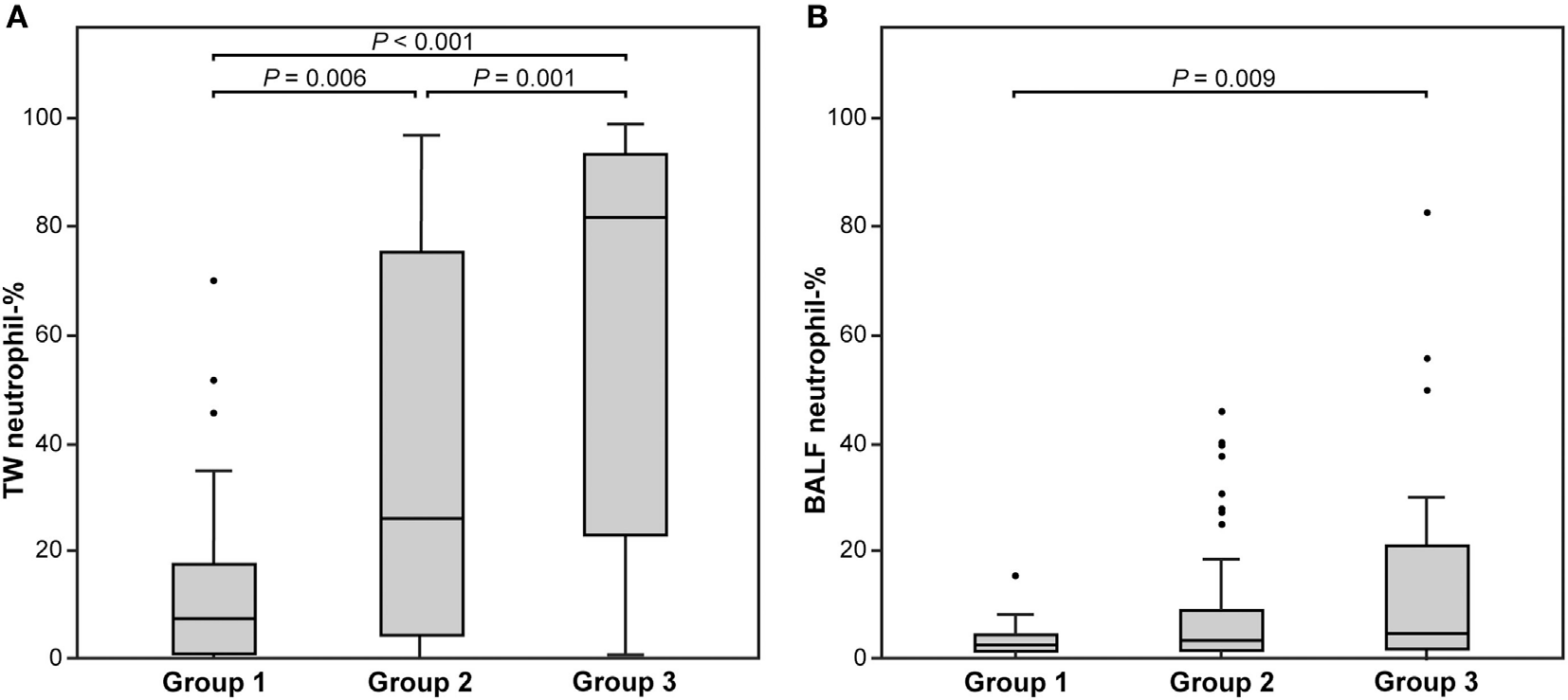
The tracheal wash [TW; **(A)**] and bronchoalveolar lavage fluid [BALF; **(B)**] neutrophil percentage among three groups of horses. Group 1 included clinically healthy control horses without signs potentially related to respiratory disease (*n* = 33). Group 2 included horses that had at least one of the following signs: cough, nasal discharge, epistaxis, fever or poor performance, and did not belong to Group 1 or Group 3 (*n* = 79). Group 3 included horses that had both cough and nasal discharge and/or highest mucus score (3/3) confirming respiratory disease (*n* = 42). The significant differences between groups (Dunn’s non-parametric comparison) are presented. Each box represents the interquartile range (25th to 75th percentiles), the central horizontal line is the median value, and the whiskers represent the range. Outliers are marked by black points.

The distributions of TW and BALF neutrophil percentage in Group 1 and Group 3 are presented in Figure 1. The neutrophil percentages in the TW and BALF samples of all three groups are presented in Figure 2. Of the other cell types, only macrophage percentages differed among groups for both TW and BALF samples, although in some horses the eosinophils and mast cells were above the reference values of 1% and 2%, respectively (Table 1). Most horses in this study had neutrophilic lung inflammation, thus the study concentrated on TW and BALF neutrophils. Detailed data on the horses and TW and BALF cytology results regarding other cell types than neutrophils are presented in Table 1.

**Table 1:** The signalement, endoscopy mucus score (0−3), blood leukocyte count, plasma fibrinogen concentration, and tracheal wash (TW) and bronchoalveolar lavage fluid (BALF) cytology in three groups of horses.

	Group 1 (*n* = 33)	Group 2 (*n* = 79)	Group 3 (*n* = 42)
Age (years); median (range)	8 (3−19)	10 (1−21)	12 (2−26)
Sex (n)MareGeldingStallion	1599	29455	16215
Endoscopy mucus score; median (range)	0 (0−2)	1 (0−2)	2 (0−3)
TW eosinophil%; median [IQR]	0.0 [0.0]	0.0 [0.4]	0.0 [0.0]
TW basophil%; median [IQR]	0.0 [0.0]	0.0 [0.0]	0.0 [0.0]
TW lymphocyte%; median [IQR]	2.0 [3.3]	3.6 [3.6]	2.0 [6.7]
TW macrophage%; median [IQR]	37.4 [49.3]	34.2 [62.2]^a^	15.4 [48.3]^a^
BALF eosinophil%; median [IQR]	0.0 [0.4]	0.0 [0.4]	0.0 [0.4]
BALF mast cell%; median [IQR]	3.0 [2.6]	2.4 [2.3]	1.4 [1.9]
BALF lymphocyte%; median [IQR]	51.4 [17.0]	44.9 [18.6]	52.7 [24.9]
BALF macrophage%; median [IQR]	41.4 [19.9]^b^	43.7 [19.5]^c^	31.1 [18.3]^b,c^
Fibrinogen (g/L; ref. < 4 g/L)	3.4 [0.6]	3.5 [1.0]	3.6 [1.3]
Total blood leukocyte count (×10^9^/L)	7.6 [1.7]	7.2 [2.1]	8.5 [3.3]

*Group 1: clinically healthy control horses without signs potentially related to respiratory disease. Group 2: horses with at least one of the following signs: cough, nasal discharge, epistaxis, fever or poor performance, and did not belong to either Group 1 or Group 3. Group 3: horses with both cough and nasal discharge and/or highest mucus score (3/3) confirming respiratory disease. All horses (*n* = 154) that had been subject to both TW and BAL sampling at rest during 2009−2015 in Helsinki University Equine Teaching Hospital were included. The significant differences between groups are presented with the letters a, b, and c and corresponding *P*-values as a footnote*.

*^a^P = 0.021*.

*^b^P = 0.002*.

*^c^P < 0.001*.

Categorized TW and BALF neutrophil cytology results of all horses (*n* = 154) that were based on the neutrophil cutoff values of 20% for TW and 5% for BALF were significantly associated with each other (Fisher’s exact *P* < 0.001), but 17.5% of the horses were classified differently by the two compared tests (Table 2). The agreement of these two tests for neutrophil cytology assessed by the kappa value was 0.652 (95% CI 0.537–0.768), which indicated substantial agreement (18). The quantity of mucus in the trachea correlated with both TW (*P* < 0.001, Spearman ρ = 0.634) and BALF (*P* < 0.001, ρ = 0.415) neutrophil percentages. Additionally, neutrophil (*P* < 0.001, ρ = 0.667), eosinophil (*P* = 0.002, ρ = 0.327), and macrophage (*P* < 0.001, ρ = 0.371) percentages correlated between TW and BALF.

**Table 2:** Cross tabulation table showing the number (%) of horses within each diagnostic class of tracheal wash (TW) and bronchoalveolar lavage fluid (BALF) cytology results of all horses (*n* = 154).

	TW > 20%	TW ≤ 20%	Total
BALF > 5%	57 (37.0%)	4 (2.6%)	61
BALF ≤ 5%	23 (14.9%)	70 (45.5%)	93

Total	80	74	154 (100%)

*Diagnostic cutoff values (neutrophil percentage of the total quantity of inflammatory cells) are set at 20% for TW and 5% for BALF*.

**Figure 3:**
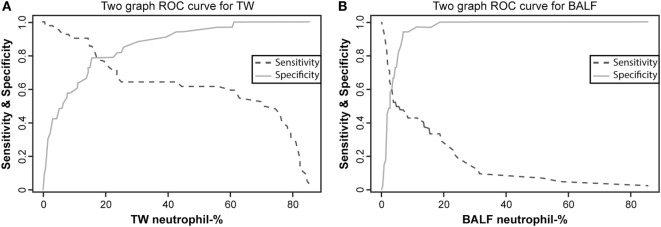
Two-graph receiver operating characteristic (ROC) curves with sensitivity and specificity plotted against the neutrophil percentage of tracheal wash [TW; **(A)**] and bronchoalveolar lavage fluid [BALF; **(B)**] for horses in Group 1 and Group 3. See Figure 1 for group definitions.

We used Group 1 and Group 3 data to compare TW and BAL neutrophil percentage results and found that the AUC of the ROCs was 0.884 (95% CI 0.810–0.958) for TW, which indicated a moderately accurate test. In comparison, the AUC of the ROCs of 0.685 (95% CI 0.565–0.805) for BAL indicated a less accurate test. The TW neutrophil percentage histogram was bimodal (Figure 1) but some obviously diseased horses, as assessed by history, physical examination, and endoscopy mucus score, had low TW neutrophil percentages. Both Se and Sp values were optimized at cutoff of 17.7% (Youden’s *J* value) where they both were approximately 80% in the two-graph ROC curve for TW neutrophil percentage (Figure 3A). It can be seen from the graph that the target Se of 90% can be reached with a neutrophil cutoff of approximately 40%, and the Sp value would decline to approximately 70%. On the other hand, the BALF neutrophil percentage histogram (Figure 1B) revealed that most of the control and the diseased horses had low values, and only a few of the diseased horses had a high percentage of neutrophils. The two-graph ROC curve for BALF neutrophil percentages (Figure 3B) for both Se and Sp revealed that they were optimized at a cutoff of 7% (Youden’s *J*) with an Sp of approximately 90% but an Se of only 50%. It can be seen from the graph that a target Se of 90% can be reached with cutoff of around 1%, whereas the Sp declined to approximately 0%. At the cutoff of 5%, the Sp is around 80%, whereas Se is only approximately 50%.

The median Se and Sp obtained by the Bayesian models for Group 3 (diseased) were 86.3% (95% probability interval 78.9–92.4) and 72.5% (95% PI: 61.5–81.8), respectively, for TW neutrophil percentages. The corresponding values for the BALF neutrophil percentages were 92.4% (95% PI: 80.1–99.5) and 58.4% (95% PI: 4.6–97.5), respectively. The median Se and Sp for Group 1 (non-diseased) were 83.0% (95% PI: 66.5–93.7) and 89.2% (95% PI: 80.3–95.4), respectively, for TW neutrophil percentages. The corresponding values for BALF neutrophil percentages were 51.5% (95% PI: 4.7–96.6) and 81.1% (95% PI: 64.7–94.6). The model with non-informative priors gave 90.5% prevalence in the diseased population (95% PI: 83.1–95.9) and 12.1% in the non-diseased population (95% PI: 2.7–27.7).

With the neutrophil cutoff values obtained from two-graph ROC curves (17.7% for TW and 7% for BALF), crosstabulation analysis of TW and BALF cytology results for Group 2 (*n* = 79) horses was performed, and the results are presented in Table 3. The TW and BALF test results for this group were significantly associated with each other (Fisher’s exact *P* < 0.001), but 19% of the horses were classified differently by the two tests (Table 2).

**Table 3:** Cross tabulation table showing the number (%) of horses within each diagnostic class of tracheal wash (TW) and bronchoalveolar lavage fluid (BALF) cytology results of Group 2 horses (*n* = 79).

	TW > 17.7%	TW ≤ 17.7%	Total
BALF > 7%	28 (35.4%)	1 (1.3%)	29
BALF ≤ 7%	14 (17.7%)	36 (45.6%)	50

Total	42	37	79 (100%)

*Diagnostic cutoff values (neutrophil percentage of the total quantity of inflammatory cells) are set to 17.7% for TW and 7% for BALF as estimated by two graph ROC curves*.

## Discussion

We compared the cytology of TW and BALF tests in horses with or without signs related to respiratory disease in this study. Only 17.5% of horses in our population had differing diagnostic interpretation of airway inflammation between the TW and BAL based on the neutrophil percentages and using the conventional cutoff values (3, 4). When only the horses with clinically less distinct clinical respiratory signs (Group 2) were assessed with the cutoff values of the ROC curves, agreement was approximately similar between the two tests. Previously, Malikides et al. (5) reported a higher disagreement of 37% in diagnostic interpretation between TW and BALF neutrophil percentages for airway inflammation diagnosis in young Australian racehorses in training presenting with poor performance. Furthermore, the agreement between the two tests in our study population that was assessed by kappa value was considered good (22) or at least substantial or moderate (18, 23) in contrast to previously published study data (24). The TW test was reported to be more sensitive in detecting airway neutrophilia by Malikides et al. (5), which agrees with our finding in the present study. However, some caution is required in making this comparison. The sampling in the studies by Malikides et al. (5) and by Allen et al. (24) was performed after high-speed treadmill exercise in contrast to our horses which had been sampled at rest: this might have affected the results since exercise is known to increase the neutrophil percentage in TW (25). Furthermore, these studies investigated only racehorses (5) or older National Hunt horses (24) in contrast to our study population of mixed breeds, and environmental conditions were different than in our study. This might have had an effect on the results.

We also investigated the currently used cutoff values of neutrophils for TW and BALF to justify their use in clinical practice for diagnosing airway inflammation. A sensitive method with unanimous cutoff value is needed in daily clinical equine practice to classify accurately the horse as being either diseased or healthy, since the diagnosis of respiratory disease in horses is often challenging due to non-specific clinical signs, such as poor performance. The current cutoff values for different cell types in BALF have recently been challenged. It has been suggested that they would be too restrictive, and new cutoff values of either >10% neutrophils, >5% mast cells, or >5% eosinophils for BALF have been proposed for diagnosis of airway inflammation (4, 8–11). Our data revealed that a cutoff of 10% for neutrophils would have resulted in an Sp of 95%, but with an Se of only 45%, thus indicating a high proportion of false-negative results. Regarding TW, the reference values for equine tracheal secretions have not been fully defined since there is large variability in the cellular content of TW samples in healthy horses (26–28). In particular, the cutoff for neutrophils still remains unclear, and there seems to be an overlap between normal horses and horses with respiratory disease (26–28). A limit of 20% of neutrophils has been conventionally used (3, 29), but there is some variation in cutoff values between different laboratories. The results of this study suggest that the current cutoff values for neutrophils, 20% for TW and 5% for BALF, would be still appropriate for use in clinical practice for the diagnosis of airway inflammation in order to achieve acceptable Se. However, the importance of TW or BALF cytology results for the final diagnosis should still always be determined in combination with the horse’s history, clinical examination and airway endoscopy findings, to improve the diagnostic accuracy (4).

Contrary to many previously published studies (5, 12–14), we found a good correlation between the neutrophil percentage of TW and BALF. A weak but statistically significant correlation was found between eosinophils and macrophages between the two methods. Based on these results and due to neutrophils, and subsequently macrophages, being the only cell types with significant difference between groups in TW and BALF, we therefore concentrated on the neutrophilic airway inflammation type in this study. In future studies, other cell types could also be assessed similarly. We found no correlation between the two methods for mast cells, nor differences between groups in this study. Mast cells are mainly distributed in the small distal airways and alveoli, and thus are a rare finding in the trachea (13). The diagnostic significance of a particular percentage of mast cells in BALF is not fully defined. A large range of 0.7–12.3% (median 9%) of mast cells in BALF has been previously reported among horses with no clinical evidence of respiratory tract disease (30). Furthermore, the quantity of mucus in the trachea correlated positively to BALF and even more strongly to TW neutrophil percentage, as found previously by some (9) but not all studies (8, 11, 31). Earlier studies have indicated that healthy horses have either no visible mucus or only a few isolated spots in their trachea (8, 31). Our results suggest that visible mucus in the trachea is likely to indicate neutrophilic airway inflammation.

Although BAL has been a standard diagnostic procedure in horses since the 1980s, there are no standardized guidelines for all steps of the sampling or cytological interpretation, and the method used is also known to have a considerable effect on the results (4). The possible sources of variation include the instilled volume of fluid, the number of aliquots used, dwelling time of fluid, lung area lavaged, aspiration of the aliquot of instilled fluid (manual or pump), handling and conservation of the samples and finally, cytological analysis of the BALF and reporting the results. According to the expert consensus regarding IAD, TW cytology is not considered an appropriate alternative to BALF cytology for the diagnosis or airway inflammation because BAL is considered to be a more sensitive and specific method to detect lower airway inflammation (4) contrary to our results. If BAL remains the recommended method of sampling, then standardization of the procedure must be implemented. However, the TW and BALF cytology of the large number of clinical cases in our study suggest that TW cytology is still at least comparable or even superior, easy, and an affordable tool in daily clinical equine practice, at least in cases of neutrophilic lung inflammation. Moreover, the Se and Sp of TW seem to be generally superior to those of BAL as analyzed by both conventional statistics and Bayesian modeling.

Some limitations of the study need to be pointed out. The horses were client-owned true clinical cases and thus lived in varying stable conditions, were managed differently, and transported to the hospital over varying distances. The environmental factors including organic dust are known to have an influence on the severity of clinical signs and airway neutrophilia (4), which might have had an effect on the results. Unfortunately, we were not able to collect this information. Moreover, the possible gender or breed differences were not considered in this study. These data were collected over several years, thus we were not able to have the same clinician performing the sampling of horses at all times. However, the TW and BALF collecting procedures are in routine use in our hospital setting, and therefore we assume minor variability in the sampling technique that was used over this time period. Moreover, a single experienced technician examined all the cytology slides. Counting 400 cells/slide is currently recommended (32); however, our samples were analyzed in a clinical diagnostic laboratory and only 300 cells/slide were counted, which might have had an effect on the results. Furthermore, the results might have been affected by a systematic error due to a single technician performing the counts. Unfortunately, automated cell counters for equine TW or BALF samples are not available. The BAL was performed in only one lung (right side) on all horses. Recently, Depecker et al. (11) suggested that BAL performed only on one lung might not be representative of the contralateral lung on the same horse. Therefore, it would be important to sample both lungs to aid the diagnosis of lower airway inflammation, especially in those cases for which the first lung’s cytology is normal. The scoring of the mucus accumulation in the trachea in this study was performed subjectively from 0 to 3 by the clinicians performing the sampling. This has been in routine use in our hospital although a more detailed scoring from 0 to 5 is recommended (33).

We conclude that TW and BALF neutrophil percentages correlated well in our patient material with naturally occurring respiratory disease of varying severity and in healthy horses indicating that either method is sufficient to detect airway neutrophilia with TW being more sensitive and specific. We also suggest that the currently used neutrophil cutoff values of 20% for TW and 5% for BALF would still be appropriate to use in clinical work for the diagnosis of airway inflammation. Our understanding of normal respiratory tract cytology is still evolving from clinical experience and research, and the most useful sampling method will be dependent on the type and the severity of the suspected disease process in the individual horse.

## Data Availability Statement

The data sets for this manuscript are not publicly available because they contain clinical patient data and the animals might be identifiable. Requests to access the data sets should be directed to Heini Rossi (heini.rossi@helsinki.fi).

## Ethics Statement

The study was carried out in accordance with the recommendations of the Finnish Animal Experiment Board (asymptomatic horses) and Viikki Ethics Committee (symptomatic horses). The protocol was approved by the Finnish Animal Experiment Board.

## Author Contributions

HR contributed to collection of the samples and data, data analysis, and preparation of the manuscript. A-MV contributed to statistical analysis of the data and preparation of the manuscript. MR contributed to study design, data analysis, and preparation of the manuscript. ER contributed to data collection. MMR contributed to preparation of the manuscript. and AM contributed to study design, collection of the samples and data, data analysis, and preparation of the manuscript.

## Conflict of Interest Statement

The authors declare that the research was conducted in the absence of any commercial or financial relationships that could be construed as a potential conflict of interest.

## Footnotes

^1^http://epitools.ausvet.com.au (accessed December 11, 2017).

^2^http://www.openbugs.net/w/FrontPage (accessed December 11, 2017).

^3^http://cadms.ucdavis.edu/diagnostictests/2dept1p.html (accessed December 11, 2017).

^4^http://rdrr.io/cran/epiR/man/epi.betabuster.html (accessed December 11, 2017).

^5^http://www.r-project.org/ (accessed December 11, 2017).

## Abbreviations

AUC, area under the curve; BAL, bronchoalveolar lavage; BALF, bronchoalveolar lavage fluid; EIPH, exercise induced pulmonary hemorrhage; ROC, receiving operating characteristic; TW, tracheal wash.
